# AFTR: A Robustness Multi-Sensor Fusion Model for 3D Object Detection Based on Adaptive Fusion Transformer

**DOI:** 10.3390/s23208400

**Published:** 2023-10-12

**Authors:** Yan Zhang, Kang Liu, Hong Bao, Xu Qian, Zihan Wang, Shiqing Ye, Weicen Wang

**Affiliations:** 1 School of Artificial Intelligence, China University of Mining and Technology-Beijing, Beijing 100083, China; tsp1600401029@student.cumtb.edu.cn (Y.Z.);; 2 College of Robotics, Beijing Union University, Beijing 100027, China

**Keywords:** 3D object detection, multi-sensor fusion, transformer, autonomous driving, misalignment

## Abstract

Multi-modal sensors are the key to ensuring the robust and accurate operation of autonomous driving systems, where LiDAR and cameras are important on-board sensors. However, current fusion methods face challenges due to inconsistent multi-sensor data representations and the misalignment of dynamic scenes. Specifically, current fusion methods either explicitly correlate multi-sensor data features by calibrating parameters, ignoring the feature blurring problems caused by misalignment, or find correlated features between multi-sensor data through global attention, causing rapidly escalating computational costs. On this basis, we propose a transformer-based end-to-end multi-sensor fusion framework named the adaptive fusion transformer (AFTR). The proposed AFTR consists of the adaptive spatial cross-attention (ASCA) mechanism and the spatial temporal self-attention (STSA) mechanism. Specifically, ASCA adaptively associates and interacts with multi-sensor data features in 3D space through learnable local attention, alleviating the problem of the misalignment of geometric information and reducing computational costs, and STSA interacts with cross-temporal information using learnable offsets in deformable attention, mitigating displacements due to dynamic scenes. We show through numerous experiments that the AFTR obtains SOTA performance in the nuScenes 3D object detection task (74.9% NDS and 73.2% mAP) and demonstrates strong robustness to misalignment (only a 0.2% NDS drop with slight noise). At the same time, we demonstrate the effectiveness of the AFTR components through ablation studies. In summary, the proposed AFTR is an accurate, efficient, and robust multi-sensor data fusion framework.

## 1. Introduction

Autonomous driving (AD) is a safety-critical task. Multi-modal sensors that are fitted to self-driving cars, such as cameras, radar, and LiDAR (light detection and ranging), are designed to enhance the accuracy and robustness of AD operations [1,2,3]. The camera captures ambient light, allowing it to obtain rich color and material information, which, in turn, provides rich semantic information. The millimeter-wave radar transmits and receives electromagnetic waves to obtain sparse orientation, distance, and velocity information from target objects. Additionally, LiDAR uses lasers for ranging, and, in AD, a multibeam LiDAR is commonly employed to perform the dense ranging of the environment, providing geometric information. To achieve advanced autonomous driving, it is crucial to fully utilize multi-sensor data through fusion methods, allowing for the integration of information from different sensors.

There are two main challenges facing current multi-sensor fusion approaches in autonomous driving. The first challenge is the heterogeneity of the data: multi-sensor data are generated from multiple sensors with different data representations, expressions (color or geometric), coordinates, and levels of sparsity; this heterogeneity poses difficulties for fusion. In most deep-learning-based fusion methods, it is necessary to align data accurately, both temporally and spatially. Additionally, during the feature fusion process, multi-source data features are obtained at different scales and from different viewpoints; this causes feature blurring and affects the accuracy of the model [4,5]. The second challenge is dynamic scene adaptation: when one of the modalities in the fusion method is disturbed, such as when adverse weather conditions, misalignment, or sensor failure is encountered, the performance of the model can be significantly reduced [6]. Many data fusion methods primarily focus on achieving state-of-the-art performance benchmarks, which only addresses one aspect of the multi-sensor fusion challenge. An ideal fusion model should possess comprehensive properties; each individual model should not fail, regardless of the presence or absence of other modalities or the integrity of other modalities, and the model should achieve improved accuracy when incorporating multi-sensor data.

In facing the challenge caused by the heterogeneity of multi-sensor data, transformer-based methods have gained significant attention in autonomous driving. Transformers establish a connection between spatial information and the features extracted from the front view (camera plane), and they are SOTA (state of the art) in 3D object detection. For example, DETR3D [7], inspired by methods like DETR [8,9], realizes end-to-end 3D object detection by constructing a 3D object query. BEVFormer [10] implements the BEV (bird’s eye view) space interaction of current and temporal image features through a spatiotemporal transformer, achieving outstanding results in 3D perception tasks. The transformer’s impressive performance in monocular image-based 3D detection tasks also allows it to implicitly capture the correlation of data between different modalities, which is particularly crucial in multi-sensor data fusion methods. Furthermore, because of the implementation of image features sampled in BEV space, there is the possibility of representing multi-sensor data under a unified space. BEVFusion [5,11] proposes a unified representation of image and point cloud data under BEV space by reconstructing the depth distributions of multi-view images in 3D space through LSS [12] and fusing them to the 3D point cloud data represented in BEV through the residual module Fusion. However, BEVFusion suffers from feature blurring in the fusion process brought about by depth estimation errors.

In facing the challenge caused by dynamic scenes, CMT [13] introduces a masked model training strategy, which improves the robustness of the model by feeding the modal failure data into the network for training. DeepFusion [14] tackles the alignment issue between point cloud and image features by leveraging a global attention mechanism, achieving an implicit alignment of the point cloud with the image in terms of features. The other methods [10,13,15], while indirectly forming an implicit alignment between multi-sensor features through a reference point, all utilize an accurate sampling of the camera’s extrinsic parameters in the projection of the reference point to the image features, which does not alleviate the problems caused by misalignment.

To address the challenge above, we propose an adaptive fusion transformer (AFTR) for 3D detection tasks—a simple, robust, end-to-end, 3D object detection framework. Firstly, we propose an adaptive spatial cross-attention (ASCA) mechanism. ASCA realizes the implicit association of 3D object queries with spatial multi-sensor features through learnable offsets, and it only interacts with the corresponding features to realize local attention. ASCA avoids the information loss caused by the 3D-2D feature projection, since ASCA can directly sample in space. Then, we propose a spatial temporal self-attention (STSA) mechanism, which equates the displacement caused by the self-ego motion and the target motion to learnable offsets. We indicate the contributions of the proposed AFTR as follows:

- To the best of our knowledge, the AFTR is the first fusion model that interacts with both 2D representational features and 3D representational features and interacts with 3D temporal information.
- The AFTR outperforms on 3D detection tasks through the cross-modal attention mechanism and the cross-temporal attention mechanism, demonstrating SOTA performance on the nuScenes dataset.
- The AFTR is the most robust framework compared to existing fusion modals; it has the smallest performance drop in the face of misalignment, and better robustness can be achieved via augmented learning using extra noisy data.

Here, we present the organization of the full paper. In Section 2, we first present the current framework for 3D object detection based on single-sensor data, followed by the current state of the art in the development of multi-sensor data fusion frameworks. In Section 3, we discuss the structure of the proposed AFTR framework in detail. In Section 4, we present the datasets used in the AFTR and the evaluation metrics for 3D object detection, and we describe in detail the setup of the AFTR in specific experiments. In Section 5, we compare the experimental results of the AFTR with those of SOTA methods and illustrate the effects of parameter settings and the components on the AFTR through a detailed ablation study, and, further, we test the robustness of the AFTR in dynamic scenes by applying noise to the alignment parameters. In Section 6, we summarize the proposed AFTR with a brief description of its advancements and limitations.

## 2. Related Works

In this section, we provide an introduction to relevant single-sensor-based (both camera-only and LiDAR-only) and fusion-based 3D object detectors. In Section 2.1, we focus on transformer-based camera-only 3D object detectors, while CNN-based methods are briefly described for the following reasons: (1) in the field of 3D object detection, transformer-based architectures have become dominant and have overwhelmed CNN-based methods in terms of performance, and (2) the proposed AFTR is a transformer-based framework, which is inspired by both image-based and the fusion method transformer frameworks. In Section 2.2, we present the relevant and most commonly used LiDAR-only 3D object detectors based on different point cloud representations. In Section 2.3, we detail the current SOTA transformer-based fusion model.

### 2.1. Camera-Only 3D Object Detector

In this section, we present only the CNN-based methods mentioned later, focusing on the transformer-based camera-only 3D detector.

#### 2.1.1. CNN-Based Method

LSS [12] introduces the lift-splat-shoot paradigm to address the bird’s-eye view perception from multi-view cameras. It involves bin-based depth prediction for lifting image features to 3D frustums, splatting these frustums onto a unified bird’s-eye view, and it performs downstream tasks on the resulting BEV feature map. FCOS3D [16] inherits from FCOS [17] and predicts 3D objects by transforming 7-DoF 3D ground truths to image view.

Since 3D target detection involves depth estimation, CNN-based methods have difficulties in modeling planar images in space, which is what the transformer excels at. In particular, after BEV-based perception methods were proposed, transformer-based frameworks outperformed CNN-based methods in the field of 3D object detection.

#### 2.1.2. Transformer-Based Method

Benefiting from the fact that transformers can establish a correlation between spatial space and image features, transformer-based camera-only detectors achieve better performance in 3D object detection tasks. These methods can be broadly categorized into object-query-based, BEV-query-based, and BEV-depth-based methods.

DETR3D [7] inherits from DETR [8], which introduces object queries and generates a 3D reference point for each query. These reference points are used to aggregate multi-view image features as keys and values, and cross-attention is applied between object queries and image features. This approach allows each query to decode a 3D bounding box for object detection. DETR4D [18] performs temporal modeling based on DETR3D, and this results in better performance. PETR [19] achieves 3D object detection by encoding 3D position embedding into 2D images to generate 3D position-aware features. PolarFormer [20] proposes a polar cross-attention mechanism based on polar coordinates, which achieves excellent detection performance under BEV. BEVDet [21] extracts features from multi-view images through LSS [12] and a BEV encoder, and it transforms them into BEV space and performs 3D object detection. BEVDet4D [22] obtains better results than BEVDet by extending BEVDet and fusing BEV features from historical and current timestamps. BEVDepth [23] continues to optimize on the basis of BEVDet and BEVDet4D by supervising and optimizing depth estimations through camera extrinsic parameters and the point cloud to achieve better results. BEVStereo [24] solves the blurring and sparsity problems caused by depth estimation in a series of methods such as BEVDet through the improvement of the temporal multi-view stereo (MVS) technique, and the improved MVS can handle complex indoor and outdoor scenes to achieve better 3D detection. BEVFormer [10] and BEVFormerV2 [25] are based on Deformable DETR [26], which interacts with image features by generating reference points in BEV, avoiding the computation of the transformation of 2D features to 3D features, and realizing robust and efficient 3D object detection. Although transformer-based camera-only frameworks have made breakthroughs in 3D object detection, they still have a reasonable performance disadvantage compared to point cloud methods or fusion-based methods that natively gain 3D geometric information.

### 2.2. LiDAR-Only 3D Object Detector

In this subsection, we briefly describe the original papers and detectors involved in commonly used LiDAR feature extraction methods. Point cloud data are usually feature-extracted under three representations: points, voxels, and pillars.

PointNet [27] pioneered the method of feature extraction directly on the raw point cloud with its MLP (multilayer perception) layers and max-pooling layers. On this basis, PointNet++ [28] achieves better performance in 3D target detection and segmentation tasks by optimizing local feature extraction.

VoxelNet [29] converts sparse point cloud data into regular stereo grids, which provides the basis for CNN implementation, and SECOND [30] improves the efficiency of feature extraction under voxel representation by employing a sparse convolution network [31]. This is currently the most commonly used feature extraction method.

PointPillars [32] extracts the pillar features of the point cloud in the longitudinal direction through PointNet, forming a particular type of regular 2D grid data with channels, which provides the possibility of using the 2D CNN method.

PointVoxel-RCNN (PV-RCNN) [33] achieves better object detection performance by fusing features under two representations (points and voxels).

Although point cloud data natively possess 3D geometric information and perform well in 3D perception, due to their sparseness, it is difficult for the point cloud to accurately detect occluded, far, and small targets.

### 2.3. Fusion-Based 3D Object Detector

F-PointNet [34] and PointPainting [4], as two typical sequential-result-level fusion models, require accurate image detection frameworks with precise multi-modal sensor calibration, and they are susceptible to wrong detection, omissions, and misalignment due to the image detector. FusionPainting [35] directly fuses the segmentation results of the LiDAR data and camera data via adaptive attention, and these are fed into the 3D detector to obtain the results. MVX-Net [36] is a feature-level fusion model, which samples and aggregates image features by projecting voxels onto the image plane, and it is also affected by misalignment.

Recently, feature-level fusion models based on transformers have become major players, benefiting from the fact that transformers can establish feature-to-feature relationships, which is important for multi-sensor data fusion. TransFusion [37] uses image features to initialize the object query; it updates the query by interacting with LiDAR features, and then it interacts with the image features and outputs the 3D detection results. DeepFusion [14], however, uses LiDAR features as the query to interact with image features, and then it updates the output features with LiDAR features and outputs the 3D detection results. DeepInteraction [38] argues that the model should learn and maintain the individual modal representations, and it proposes that LiDAR and camera features should interact with each other in order to fully learn the features of each modality. BEVFusion [5,11] proposes a simple and efficient framework to predict the depth distribution of multi-view images using LSS [12], represent the image features under BEV, and subsequently generate fusion features by aggregating the BEV LiDAR features and BEV camera features through the BEV encoder to alleviate the feature blurring between multi-sensor data features. UVTR [39] avoids the loss of information caused by compression into BEV space by proposing to represent both the image and the point cloud in voxel space. FUTR3D [15] and CMT [13], however, generate 3D reference points through object queries and use 3D reference point sampling or interaction with multi-modal features to update the object queries, and then they perform 3D target detection through a transformer-based decoder. However, both FUTR3D and CMT use calibration parameters to achieve the direct exact matching of multi-sensor data, which is detrimental to robustness.

## 3. AFTR Architecture

**Figure 1:**
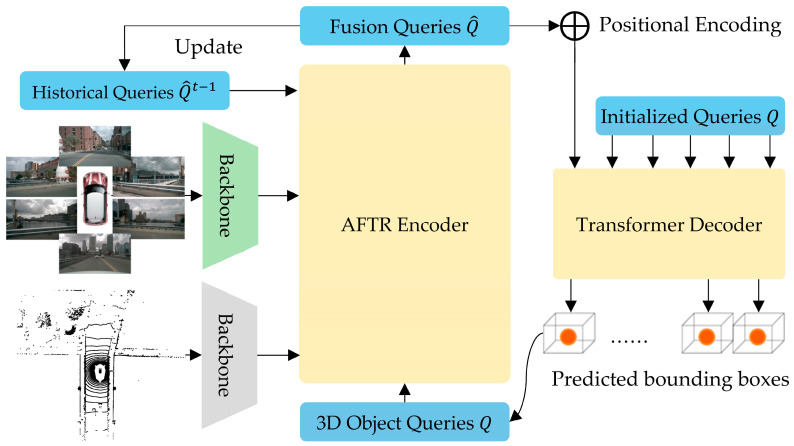
The overall framework of AFTR.

**Figure 2:**
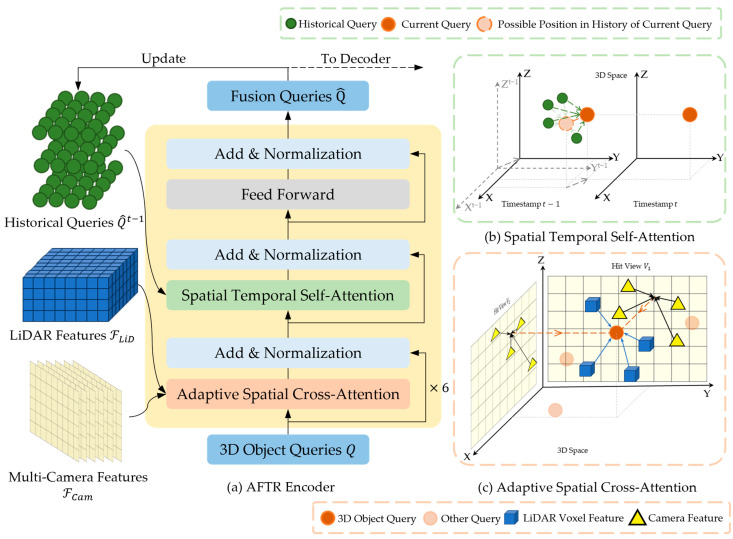
Detailed structure of AFTR encoder.

In this paper, we propose the AFTR (adaptive fusion transformer), which implicitly aligns the features of multi-sensor data to achieve more robust 3D object detection results. The AFTR can be divided into four parts, as shown in Figure 1. The AFTR takes the multi-view camera data and LiDAR data as input data and extracts features through individual backbones (Section 3.1). At the same time, the fusion queries of the historical timestamp ${\hat{Q}}^{t-1}$ are also input into the AFTR encoder. The randomly generated 3D object queries Q interact with the features of the multi-sensor data, and the historical information is finally updated with the fusion queries $\hat{Q}$ of the current timestamp. Then, the fusion queries $\hat{Q}$ are position-encoded and input into the DETR3D [7] and Deformable DETR [26] transformer decoders (Section 3.4). The fusion queries $\hat{Q}$ interact with the initialized 3D object queries Q through layer-by-layer refinement in the transformer decoder, which finally outputs the 3D object detection results. The proposed AFTR has two main components, as shown in Figure 2a: the adaptive spatial cross-attention (ASCA) module (Section 3.2) and the spatial temporal self-attention (STSA) module (Section 3.3). The input data of ASCA comprise multi-camera features ${ℱ}_{Cam}$ and LiDAR features ${ℱ}_{LiD}$ represented by voxels, and the input data of STSA comprise 3D representations of the historical frame fusion queries ${\hat{Q}}^{t-1}$. Finally, the fusion queries $\hat{Q}$ are output through the feed-forward module and used for 3D object detection.

### 3.1. Feature Extraction

The proposed AFTR learns features from multi-view images and the point cloud, and any feature extraction method that can be used on images or the point cloud can be employed in our framework.

For multi-view images, $ℐ={\left\{{ℐ}_{i}\in {\mathbb{R}}^{3\times H\times W}\right\}}_{i=1}^{n}$, where H, W, and n are the height, width, and the number of views of the image, respectively. We follow previous work [7,10,13,15,16] using ResNet [40] or VoVNet [41] for feature extraction and use FPN [42] to output multi-scale features, denoted as ${ℱ}_{Cam}^{i}={\left\{{ℱ}_{Cam}^{ij}\in {\mathbb{R}}^{C\times H_{j}\times W_{j}}\right\}}_{j=1}^{m}$ for the i-th image view with m scales, where C is the channel size of the feature, and $H_{j}$ and $W_{j}$ denote the height and width of the j-th scale features, respectively.

For the point cloud, we use VoxelNet [29] for feature extraction, and we follow FUTR3D [15] to output multi-scale voxel features by using FPN [42]. It should be noted that the point cloud features extracted in our method are represented in 3D space instead of being projected into BEV space [13,15], and the point cloud features can be denoted as ${ℱ}_{L}={\left\{{ℱ}_{L}^{j}\in {\mathbb{R}}^{C\times X_{j}\times Y_{j}\times Z_{j}}\right\}}_{j=1}^{m}$, where $X_{j}$, $Y_{j}$, and $Z_{j}$ are the sizes of the 3D voxel feature.

### 3.2. Adaptive Spatial Cross-Attention

Adaptive spatial cross-attention (ASCA) is a critical component of the AFTR, and it aims to fuse multi-sensor features while achieving implicit alignment by interacting with multi-view, multi-scale image features and 3D point cloud features through an object-query-based cross-attention mechanism. A schematic diagram of the ASCA module is shown in Figure 2c. The detection head for the AFTR is a set of object queries $Q\in {\mathbb{R}}^{C\times X\times Y\times Z}$, which has a number of $N_{ref}$ 3D object queries named $Q_{p}\in {\mathbb{R}}^{1\times C}$, where $Q_{p}$ corresponds to a reference point p=(x,y,z) in real-world 3D space. Considering the handling of multi-scale features, we normalize the 3D reference point coordinates, giving $p\in {\left[0,1\right]}^{3}$. ASCA dynamically updates each query $Q_{p}$ by interacting with and fusing multi-sensor data features.

#### 3.2.1. Interaction with Multi-View Image Features

ASCA uses the Deformable DETR [26] idea to produce an interaction between the query and multi-sensor data features for two reasons: first, the 3D reference point corresponds to only a few features, and the native attention [9] mechanism requires a query to interact with all the features, which results in extreme computational costs. Deformable DETR, by adding an offset, focuses on only query-related features. Second, determining how to find the reference point in an image is a big challenge. Previous approaches directly project the 3D reference point onto the corresponding image plane using calibration parameters, which is not robust. ASCA learns the 3D reference point to correctly associate the features by using the offset to achieve implicit alignment. We follow the hit view $V_{hit}$ in BEVFormer [10] and project the 3D reference points onto BEV to determine their possible projected view $V_{hit}=\left\{V_{i}\right\}$. Ultimately, an interaction with the features in $V_{hit}$ is achieved through ASCA. The adaptive spatial cross-attention process with image features can be formulated as Equation (1):

(1) $$ASCA_{Cam}\left(Q_{p},{ℱ}_{cam}\right)=\frac{1}{\left|V_{hit}\right|}\sum_{i\in V_{hit}}\sum_{j=1}^{m}DeformAttn\left(Q_{p},\;T_{i}\left(p\right),{ℱ}_{Cam}^{ij}\right),$$

where $Q_{p}$ is the 3D object query, m denotes the number of scales, ${ℱ}_{Cam}^{ij}$ represents the image feature of the j-th scales in the i-th view, and $T_{i}\left(p\right)$ is the project function that transforms the 3D reference point p to the i-th image plane. $T_{i}\left(p\right)$ can be represented as Equation (2):

(2) $$T_{i}\left(p\right)=T_{i}\left(x,y,z\right),\;\mathrm{where}\;\left[\begin{array}{cccc} u_{i} & v_{i} & d_{i} & 1 \end{array}\right]=\left[\begin{array}{cccc} x & y & z & 1 \end{array}\right]\left[\begin{array}{cc} R_{i}{}^{T} & 0\\ T_{i} & 1 \end{array}\right]{\left[\begin{array}{cc} CI_{i} & 0\\ 0 & 1 \end{array}\right]}^{T},$$

where $u_{i}$ and $v_{i}$ denote the normalization coordinate positions of the width and height in the i-th image plane, respectively; $d_{i}$ is the depth of the pixel, which is not used in our method; $R_{i}\in {\mathbb{R}}^{4\times 4}$ and $T_{i}\in {\mathbb{R}}^{1\times 3}$ denote the LiDAR to the i-th camera transformation matrix of rotation and translation, respectively; and $CI_{i}\in {\mathbb{R}}^{3\times 3}$ represents the i-th camera intrinsic parameters.

Following Deformable DETR, the features obtained through the offset are calculated using bilinear interpolation [43] from the four closest pixels.

In general, ASCA only interacts with the hit view image features corresponding to the object query to reduce computation. While ASCA employs camera extrinsic parameters to project 3D reference points onto the image, which only serves as a reference for sampling, ASCA uses dynamically updating offsets to implicitly align the reference points with the image features so that the object query only interacts with the related features.

#### 3.2.2. Interaction with Point Cloud Features

Since point cloud features are natively represented in 3D space, indicating the geometric features of an object in a real-world space, 3D reference points can interact with point cloud features without projection. However, the point cloud coordinates deviate from the real-world coordinates or the ego coordinates in the following cases: first, when the sensor position is translated or rotated and, second, when there is a delay due to the sampling frequency of the LiDAR. ASCA can better learn such deviations to ensure an accurate implicit alignment. The adaptive spatial cross-attention process with point cloud features can be formulated as Equation (3):

(3) $$ASCA_{LiD}\left(Q_{p},{ℱ}_{LiD}\right)=\sum_{j=1}^{m}DeformAttn\left(Q_{p},\;p,{ℱ}_{LiD}^{j}\right).$$

The offsets of the reference point are generated in 3D space, the point cloud is encoded as stereo grids regularly arranged spatially, and then the offset is located within a certain stereo grid. We express the j-th scale point cloud features corresponding to the offsets ${ℱ}_{LiD-offset}^{j}$ as Equation (4):

(4) $${ℱ}_{LiD-offset}^{j}={\left\{{ℱ}_{LiD}^{j}\left(round\left(p+\Delta_{LiD}^{jk}\right)\right)\right\}}_{k=1}^{N_{offset}},$$

where $\Delta_{LiD}^{jk}\in {\mathbb{R}}^{1\times 3}$ denotes the k-th offset in the j-th scale point cloud feature, and $N_{offset}$ is the number of offsets. We obtain the index of the 3D grid by rounding up the offset.

#### 3.2.3. Multi-Model Fusion

After obtaining the results of the $Q_{p}$ interaction with multi-view images and point cloud features, we fuse them and update $Q_{p}$. First, we concatenate the results of the ASCA interaction with the multi-sensor data and encode them using an MLP network; the process can be described as Equation (5):

(5) $$ASCA\left(Q_{p},{ℱ}_{cam},{ℱ}_{LiD}\right)=MLP\left(ASCA_{Cam}\left(Q_{p},{ℱ}_{cam}\right)⊗ASCA_{LiD}\left(Q_{p},{ℱ}_{LiD}\right)\right).$$

Finally, we update the object query $Q_{p}$ using Equation (6):

(6) $$Q_{p}=Q_{p}+ASCA\left(Q_{p},{ℱ}_{cam},{ℱ}_{LiD}\right).$$

### 3.3. Spatial Temporal Self-Attention

The incorporation of temporal information has been demonstrated to be beneficial for camera-only 3D object detection [10,18,22,44], which is still valid in multi-sensor data fusion models.

Features or queries on historical timestamps rather than the current timestamp introduce two problems: first, the misalignment of the coordinate system due to self-ego motion and, second, the misalignment of the features or query due to the motion of the object. BEVDet4D [22], BEVFormer [10], and DETR4D [18] perform the transformation between different timestamps by means of self-vehicle motion. When facing the case of object motion, BEVFormer predicts the offset in Deformable DETR [26] from the current frame queries and aggregates features in historical frames, which makes it challenging to align each object query with its own historical query. DETR4D globally interacts with queries from different timestamps by performing multi-head attention [9] to achieve the aggregation of relevant features, which also induces significant computational costs.

We propose spatial temporal self-attention (STSA), as shown in Figure 2b. Following Deformable DETR [26], STSA realizes the implicit alignment of object and historical object features by sampling and interacting with historical 3D object queries $Q^{t-1}$ and finding the specific queries $\left\{Q_{p}^{t-1},p\in \left[1,N_{ref}\right]\right\}$ associated with the current timestamp $Q_{p}^{t}$ by dynamically updating the offsets, which effectively counteracts the misalignment caused by both self-ego motion and object motion. STSA can be expressed as Equation (7):

(7) $$STSA\left(Q^{t-1},Q_{p}^{t}\right)=DeformAttn\left(Q_{p}^{t},\;p,Q^{t-1}\right),$$

where p is the 3D reference point corresponding to the current timestamp object query $Q_{p}^{t}$; notice that the offset ${\left\{\Delta_{Tem}^{k}\in {\mathbb{R}}^{1\times 3}\right\}}_{k=1}^{N_{offset}}$ is represented in 3D space.

Finally, we update the object query $Q_{p}$ using Equation (8):

(8) $$Q_{p}=Q_{p}+STSA\left(Q^{t-1},Q_{p}^{t}\right)$$

### 3.4. Detection Head and Loss

We design a learnable end-to-end transformer-based 3D detection head based on the 2D detector Deformable DETR [26], which implements the object query used for detection through L layers of the deformable attention blocks. Specifically, we use the AFTR-generated fusion features as inputs to the decoder to interact with the predefined object query, update all object queries $\hat{Q}$ at the output of each decoder layer, and predict the updated 3D reference point $\hat{p}$ by using the sigmoid function as a learnable linear projection from the updated ${\hat{Q}}_{p}$, as shown in Equation (9):

(9) $$\hat{p}=Linear\left({\hat{Q}}_{p}\right).$$

The detector finally predicts the 3D bounding box $\hat{b}$ and classification $\hat{c}$ of the object after two feed-forward network (FFN) layers, which can be expressed as Equation (10):

(10) $$\hat{b}=FFN_{reg}\left(\hat{Q}\right),\hat{c}=FFN_{cls}\left(\hat{Q}\right).$$

Finally, for the prediction of the set, the Hungarian algorithm is used to find a bipartite match between the predicted truth and the ground truth. We use Gaussian focal loss [45] for classification and L1 loss for 3D bounding box regression, and then we represent the 3D object detection total loss as Equation (11):

(11) $$ℒ=\omega_{1}{ℒ}_{reg}\left(b,\hat{b}\right)+\omega_{2}{ℒ}_{cls}\left(c,\hat{c}\right),$$

where $\omega_{1}$ and $\omega_{2}$ are the coefficients of the individual cost, and b and c are the ground truth of the 3D bounding box and the classification of the set, respectively.

## 4. Implementation Details

In this section, we focus on the experimental setup (Section 4.3) used for the training and testing of the proposed AFTR on a publicly available dataset, nuScenes (Section 4.1), as well as the metrics (Section 4.2) of the 3D object detection task.

### 4.1. Dataset

We trained and tested the AFTR on the widely used nuScenes dataset [46]. nuScenes contains multi-sensor data of 1000 scenes in Singapore and Boston, with each scene spanning 20 s and annotated with 40 keyframes (every 0.5 s). nuScenes divides these scenes into training, validation, and test sets, which contain 700, 150, and 150 scenes, respectively. For the 3D detection task, nuScenes provides annotations for 10 categories. We mainly used multi-view cameras and LiDAR for 3D object detection. The nuScenes data cover the whole environment and were acquired through six cameras at 12 FPS and 32-beam LiDAR at 20 FPS. We transformed the unlabeled point cloud of the previous nine frames to the current frame based on common practice [13,15].

Multi-modal sensor registration is an important prerequisite for data fusion. For spatial alignment, nuScenes provides the external parameters of all sensors from which we can calculate the calibration parameters across modal sensors. For time synchronization, nuScenes provides good time-synchronized multi-modal sensor data to control the camera exposure by setting triggers at specific phases (center of camera’s FOV) of the lidar rotation.

### 4.2. Metrics

In this paper, we use the nuScenes [46] official metrics to evaluate the performance of the AFTR, including the mean average precision (mAP) [47] and five types of true-positive (TP) metrics, which are better when smaller: the mean average translation error (mATE), mean average scale error (mASE), mean average orientation error (mAOE), mean average velocity error (mAVE), and mean average attribute error (mAAE). Finally, the nuScenes detection score (NDS) summarizing the above metrics can be calculated as Equation (12):

(12) $$\mathrm{NDS}=\frac{1}{10}\left(5\times \mathrm{mAP}+\sum_{\mathrm{mTP}\in \left\{\mathrm{TP}\right\}}\left(1-\min(1,\mathrm{mTP}\right)\right),$$

where {TP}={mAP, mATE, mASE,mAOE,mAAE}.

For the commonly used mAP evaluation metrics in 3D target detection tasks, they can be expressed as Equation (13):

(13) $$\mathrm{mAP}=\frac{1}{\left|C\right|\left|D\right|}\sum_{c\in C}\sum_{d\in D}AP_{c,d},$$

where C and D∈{0.5,1,2,4} are the detection classification and matching thresholds, respectively, and AP is the average precision [47,48].

### 4.3. AFTR Setup

#### 4.3.1. Feature Extraction Settings

For multi-view images, the input single image is resized to 1600 × 640. We employed ResNet-101 [40] pre-trained on FCOS3D [16] and VoVNet-99 [41] pre-trained on DD3D [43] as image feature extractors, which are the most commonly used image feature extractors in current SOTA methods [10,18,19,26], and we discuss the effect of different image feature extractors on the AFTR in the Ablation Studies Section (Section 5.2.1). Then, we used FPN to output the multi-scale features containing m=4 scales. The feature maps are sized to be 1/8, 1/16, 1/32, and 1/64 of the original features, and the channel C is 256. The use of the FPN setup is also common practice in transformer-based methods [10,13,15].

For point clouds, we set the voxel size to s=0.075 m×0.075 m×0.2 m, as we obtained the best performance at this voxel size (Section 5.2.2), and we fed them to the voxel feature extractor (VFE) and then created multi-scale point cloud features based on the FPN [42] concept with m=4 scales. We used VoxelNet [29] with sparse convolution [30] as VFE without pre-training, and the output channel C=256. The region of interest (ROI) of the point cloud is in the range of [−54.0 m, 54.0 m] along the X and Y axes, and it is in the range of [−5.0 m, 3.0 m] along the Z axis; most of the denser point clouds are contained in this range, and it is also the range of ROI of 3D space.

#### 4.3.2. Model Settings

We predefined the 3D object queries $Q\in {\mathbb{R}}^{C\times X\times Y\times Z}$ with channel C=256 and X, Y, and Z normalized in 3D ROI space. The number of $N_{ref}=900$ 3D object queries is initially distributed uniformly in ROI. ASCA contains six layers of transformer-based encoders and continuously refines the 3D object queries in each layer. For each object query, when the ASCA and STSA modules are implemented through deformable attention [26], $N_{offset}=4$ offset points correspond to the default setting in Deformable DETR [26]. Our detection head contains L=6 layers of transformer-based decoder blocks. We used the model with VoVNet-99 as the image feature extractor as the default and denoted it as AFTR.

#### 4.3.3. Training Phase

We used the open-source mmdetection3d (version 1.0.0rc6) to build the proposed model. The proposed AFTR was trained with a batch size of 1 on 1 RTX4090 GPU with 24 GB memory. The AFTR was trained with 40 batches using AdamW [49] with an initial learning rate of $2\times 10^{-5}$ and by following the cycle learning rate policy. Following prior works [7,15], $\omega_{1}$ and $\omega_{2}$ were set to 0.25 and 2.0, respectively.

For the processing of temporal information, we followed BEVFormer [10], and for each current timestamp, we randomly sampled one historical query from the previous two seconds of data, which are cached in the previous computation and do not need to be recomputed. For the computation sequence without historical data, we used self-attention [9] to compute the result in the STSA step.

In addition, in order to enhance the robustness of the AFTR in the face of misalignment due to various reasons, we added alignment noise according to BEVFormer [10] during the training phase to enable the model to learn misaligned multi-sensor data, denoted as AFTR-a.

## 5. Results and Analysis

In this section, we focus on making a comparison of the AFTR with various SOTA methods using the nuScenes dataset [46] (Section 5.1), and we explore the effects of each component of the AFTR through ablation studies (Section 5.2). Finally, we investigate the robustness of the AFTR in the face of misalignment (Section 5.3).

### 5.1. State-of-the-Art Comparison

**Figure 3:**
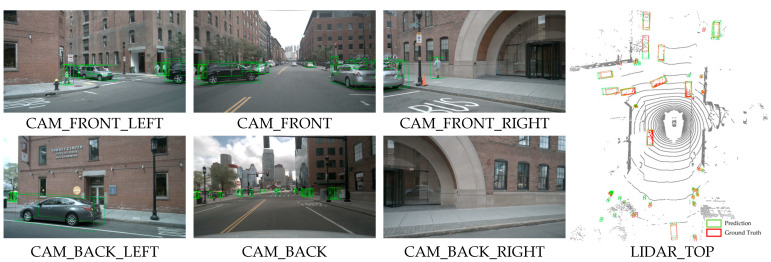
Qualitative results of AFTR for 3D object detection on the nuScenes dataset. Thanks to AFTR’s use of cross-modal attention and cross-temporal attention, the target occluded by the black car in CAM_FRONT and the smaller, more distant targets in CAM_BACK are both correctly detected.

We conducted experiments on the nuScenes dataset [46] and observed outperformance in the 3D object detection task. Quantitative results on the nuScenes test set are shown in Table 1. We set up AFTR-C, AFTR-L, and AFTR as models trained using camera data, LiDAR data, and fused data, respectively. In comparison with the camera-only model, the AFTR achieved nearly SOTA performance (0.9% to the best). In comparison with the LiDAR-only model, AFTR-L outperformed all fusion models trained with LiDAR data only, obtaining 74.9% NDS and 73.2% mAP. In comparison with the fusion model, the AFTR still achieved the best mAP and NDS without using additional enhancements (e.g., the CBGS [50] strategy or test-time augmentation). In comparisons of the AFTR series, the NDS of the AFTR improved by 33.8% compared to that of AFTR-C when fusing LiDAR data and by 4.5% compared to that of AFTR-L NDS when fusing camera data. Similarly, as shown in Table 2, the AFTR leads in the comparison of NDS and map on the nuScenes validation set. Figure 3 illustrates the qualitative results of the AFTR on the nuScenes dataset. Benefiting from the accurate multi-sensor fusion model and the incorporation of temporal information, the AFTR achieves accurate detection, even for targets with only one or two points in the point cloud.

We attribute the good performance of the proposed AFTR to two points: the first is the accurate and efficient fusion of multi-sensor data using the ASCA module, and the second is the use of the STSA module to interact with the historical data as a complement to the current timestamp data, which alleviates part of the object occlusion problem.

### 5.2. Ablation Studies

In this section, we reveal the effect of each component in the proposed AFTR through ablation studies, and the experiments in this section were all performed on the nuScenes validation set. As the AFTR is a multi-sensor data fusion model, we explore (1) the effect of the input image size and the image feature extractor on the AFTR (Section 5.2.1); (2) the effect of the size of the point cloud converted to voxels on the AFTR (Section 5.2.2); (3) the effect of the representation of point cloud features on the AFTR (Section 5.2.3); (4) the effect of temporal information on the AFTR (Section 5.2.4); and (5) the effect of the number of offsets $N_{offset}$ on the AFTR (Section 5.2.5).

#### 5.2.1. Effect of Image Size and Backbone

Complying with various leading camera-only methods and fusion methods, we resized the original images to 800 × 320 and 1600 × 640 and input them into the network for training. In Table 3, it is easy to see that the AFTR performs better when the input image size is larger, which improves NDS by 3.6% and mAP by 6.5% when compared with the smaller input image size.

We chose the current leading and more effective backbones, ResNet [40] and VoVNet [41], as the multi-view image feature extractors for the AFTR. Specifically, in the ablation study, we compare the effectiveness of ResNet-50, ResNet-101, and VoVNet-99 in 3D object detection, as shown in Table 4, which shows that VoVNet-99 obtains the best results with 73.5% NDS and 70.4% mAP.

#### 5.2.2. Effect of Voxel Size

The point cloud contains discrete, disorganized, and irregularly sparse 3D data, so voxelizing the point cloud into regular data is a better choice for perception tasks, but the voxel size affects the fineness of the geometric information and the computational complexity, which, in turn, affects the quality of the model. Here, we explore the effect of three voxel sizes on the AFTR, including 0.075 m, 0.1 m, and 0.125 m voxel units. As shown in Table 5, when the voxel is smaller and the geometric information is finer, the AFTR can obtain better results, but the reduction in the voxel size causes a $O\left(n^{3}\right)$ increase in computational complexity, so we adopt the common practice and set the voxel size to 0.075 m × 0.075 m × 0.2 m.

#### 5.2.3. Effect of LiDAR Feature Representation

In recent methods [1,13,15], the point cloud features are transformed in BEV, which requires the pooling or flattening of voxels along the z axis, leading to a loss of geometric information. In the AFTR, the 3D object queries interact directly with the voxels, which ensures the integrity of the spatial information. Here, we reveal which representation achieves better performance in the AFTR. It should be noted that, after transforming the point cloud features to BEV, the sampling and interaction of the features via ASCA are consistent with those used to obtain the image features, which are all performed in 2D space. As shown in Table 6, the AFTR obtains better performance with the 3D representation with finer geometric information.

#### 5.2.4. Effect of Spatial Temporal Self-Attention

While many approaches have demonstrated the gain of temporal information in perception tasks [10,18,22], we conducted an ablation study of the effect of STSA on the AFTR. We used the AFTR-s model without temporal information to make a comparison with the default AFTR. Specifically, in AFTR-s, the STSA module is replaced with a vanilla self-attention [9] module, and the updated query is obtained by interacting with itself through the input query. The results of the ablation study are shown in Table 7. Without temporal information, the resulting NDS and mAP of AFTR-s drop by 6.0% and 5.8%, respectively, compared to those of the default AFTR.

#### 5.2.5. Effect of Number of Offsets

The core concept of the proposed AFTR is to achieve local attention through deformable attention [26], where the query only interacts with the relevant features around the reference point, which not only saves computational costs but also achieves an implicit alignment of multi-sensor data features through 3D reference points. Deformable attention searches for the relevant features through learnable offsets, and the number of offsets $N_{offset}$ can impact the performance of the AFTR. Here, we explore the effect of $N_{offset}$ on the AFTR by setting different numbers of offsets, and, furthermore, we replace deformable attention with vanilla attention [9] to implement global attention to make a comparison with local attention. When $N_{offset}$ = 0, the query interacts directly with the reference point. The results of the ablation study of the effect of the number of offsets on the AFTR are shown in Table 8, where the AFTR achieved the best results when $N_{offset}$ = 4. It is worth noting that the use of global attention does not yield better results, while it results in a significant rise in computation. In addition, the inclusion of offsets has an impact on the robustness of the model, which we address in Section 5.3.

### 5.3. Robustness of AFTR

Although the proposed AFTR also uses the calibration parameters of multi-modal sensors, instead of directly associating features by searching for exact projection relations [13,15], we implemented a local attention mechanism by searching for corresponding features around the projection point through learnable offsets, which can mitigate the rapid degradation of performance due to timing, localization, and dynamics bias and provide a reliable robustness for AFTR in misalignment situations.

**Figure 4:**
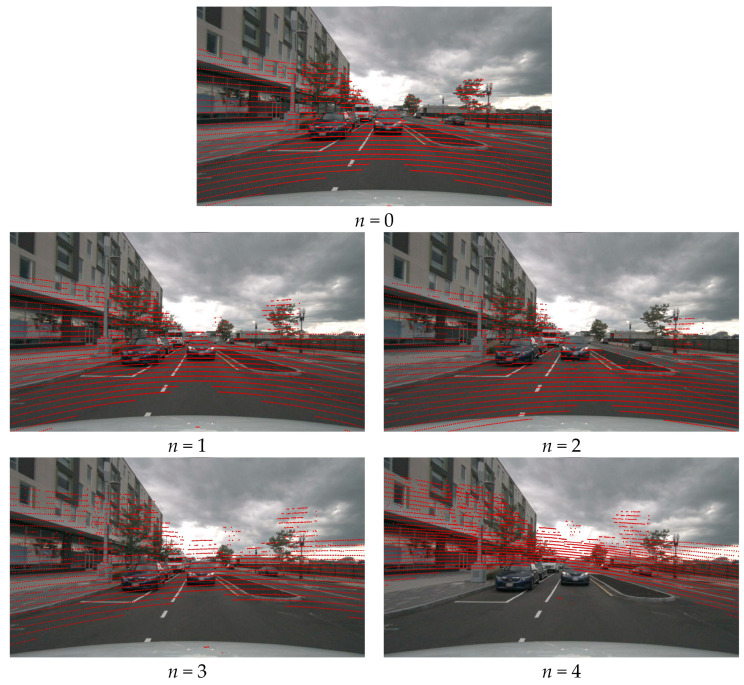
Visualization results when adding noise to multi-sensor calibration parameters. We projected the point cloud according to the calibration parameters and displayed it in the image using the red points.

Specifically, we used noise levels *n* to impose interference on the alignment parameters (or camera extrinsic parameters) of both the training and test data. Figure 4 shows the visualization results when noise is added to the multi-sensor calibration parameters. Following BEVFormer [10], for *n* levels of noise, we used normally distributed sampling to interfere with the alignment parameters, where the sampling for translations and rotations has a mean equaling 0 and a variance equaling 5*n*, and a mean equaling 0 and a variance equaling *n*, respectively. We trained and tested AFTR, FUTR3D, and BEVFormer on noisy data to observe their robustness to misalignment. Specifically, we used Delta to evaluate the accuracy of the model for misalignment, which can be described as Equation (14):

(14) Delta=1−NDSn=4NDSn=0,

where $NDS_{n=0}$ denotes the NDS under noise level *n* = 0, and so on.

In the control group, we used AFTR-s to assess the effect of temporal information on misalignment, we assessed data augmentation in the model using AFTR-a and AFTR-sa, and we assessed the robustness of global attention using AFTR-sg. Furthermore, the comparison with FUTR3D and FUTR3D-a reflects the robustness of the AFTR model.

**Figure 5:**
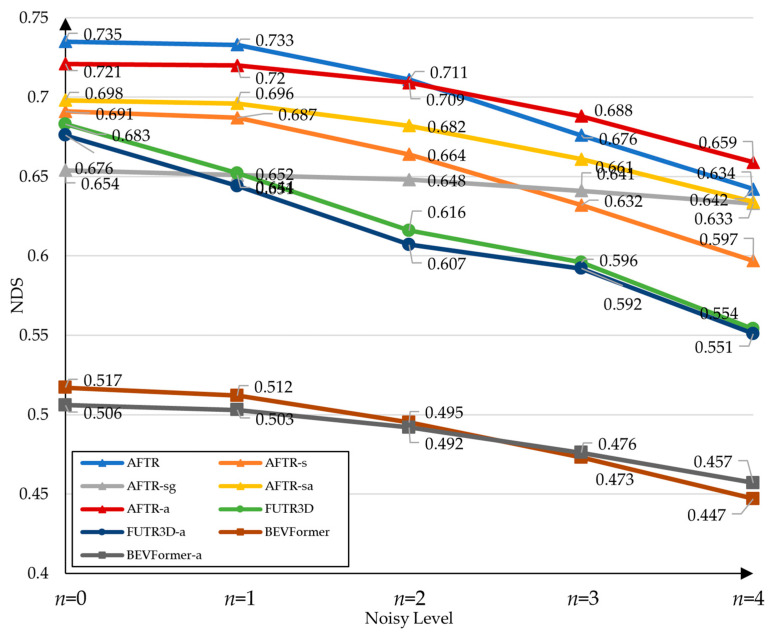
NDS results for AFTR with different calibrations of noise parameter, where *n* is the noise level. The default AFTR has a strong robustness, with only 0.2% NDS degradation at *n* = 1, while FUTR3D has 4.5% NDS degradation. In AFTR-a trained on noisy data, the NDS performance exceeds that of the default AFTR method in the presence of severe noise.

As shown in Figure 5 and Table 9, FUTR3D samples image features by projecting exact projections, resulting in a rapid degradation of the model’s performance after adding noise. With light noise *n* = 1, FUTR3D’s NDS drops by 4.5%, while the AFTR’s NDS drops by only 0.2%, already demonstrating strong robustness to misalignment. Moreover, in hard noise, the performance of FUTR3D significantly decreases by 18.9%, while the performance of the AFTR only decreases by 12.7%. Due to the exact sampling mode of FUTR3D, no robustness improvement is realized in FUTR3D-a with the addition of noise training, which has a Delta of 18.5%. For AFTR-a and AFTR-sa, the robustness is further improved compared to that of the AFTR and AFTR-s, the Delta is improved by 4.1% and 4.5%, and the performance of AFTR-a exceeds that of AFTR when the noise is large. In the comparison between AFTR-s and AFTR, we find that the addition of temporal information helps the model to be more robust to misalignment. In AFTR-sg, the model with global attention is minimally affected by misalignment because no calibration parameters are used for local attention computation, and Delta = 3.2% when facing hard noise.

## 6. Conclusions

In this paper, we proposed a transformer-based end-to-end multi-modal fusion 3D object detection framework, named the adaptive fusion transformer (AFTR). The AFTR achieves an implicit alignment of cross-modal features and cross-temporal features by adaptively sampling and interacting with multi-sensor data features and temporal information in 3D space via adaptive spatial cross-attention (ASCA) and spatial temporal self-attention (STSA) for accurate and efficient 3D object detection. Our experiments on the nuScenes dataset demonstrated that the AFTR achieves better performance by fusing multi-sensor features and improves the detection of occlusions and small targets by acquiring temporal information. In addition, when studying the AFTR in terms of the misalignment problem, we found that the AFTR has a strong robustness to minor misalignments caused by various reasons, benefiting from the abilities of adaptive correlation features.

While the proposed AFTR has many advantages, there are still some limitations. First, the current transformer-based models are more computationally intensive than the CNN-based models, and a feasible solution is to reduce the number of queries by making them mainly focus on the foreground. Second, when faced with sensor failures or distorted sensor data, the performance of the default AFTR will degrade or even be inferior to that of AFTR-L or AFTR-C trained on data from a single sensor; a possible solution to this is to incorporate failures and distortions into the training to make the model more robust.

## Figures and Tables

**Table 1:** Comparison of AFTR with various SOTA methods on nuScenes test set. Abbreviations: C is cameras, L is LiDAR, and LC is LiDAR and cameras. “FUTR3D-C” denotes a model trained and tested using only camera data, and so on.

Method	Modality	NDS	mAP	mATE	mASE	mAOE	mAVE	mAAE
DETR3D [7]	C	0.479	0.412	0.641	0.255	0.394	0.845	0.133
BEVDet4D [22]	C	0.569	0.451	0.511	0.241	0.386	0.301	0.121
BEVFormer [10]	C	0.569	0.481	0.582	0.256	0.375	0.378	0.126
PETR [19]	C	0.504	0.441	0.593	0.249	0.383	0.808	0.132
FUTR3D-C [15]	C	0.479	0.412	0.641	0.255	0.394	0.845	0.133
CMT-C [13]	C	0.481	0.429	0.616	0.248	0.415	0.904	0.147
CenterPoint [51]	L	0.673	0.603	0.262	0.239	0.361	0.288	0.136
TransFusion-L [37]	L	0.702	0.655	0.256	0.240	0.351	0.278	0.129
UVTR-L [39]	L	0.697	0.639	0.302	0.246	0.350	0.207	0.123
FUTR3D-L [15]	L	0.699	0.653	0.281	0.247	0.368	0.253	0.124
CMT-L [13]	L	0.701	0.653	0.286	0.243	0.356	0.238	0.125
PointPainting [4]	LC	0.610	0.541	0.380	0.260	0.541	0.293	0.131
FusionPainting [35]	LC	0.716	0.681	**0.256**	0.236	0.346	0.274	0.132
TransFusion [37]	LC	0.717	0.689	0.259	0.243	0.359	0.288	0.127
UVTR [39]	LC	0.711	0.671	0.306	0.245	0.351	0.225	0.124
BEVFusion [5]	LC	0.729	0.702	0.261	0.239	0.329	0.260	0.134
DeepInteraction [38]	LC	0.734	0.708	0.257	0.240	0.325	0.245	0.128
FUTR3D [15]	LC	0.721	0.694	0.284	0.241	0.310	0.300	0.120
CMT [13]	LC	0.741	0.720	0.279	**0.235**	**0.308**	0.259	**0.112**
AFTR-C	C	0.560	0.465	0.584	0.251	0.364	0.410	0.118
AFTR-L	L	0.717	0.663	0.265	0.241	0.343	**0.186**	0.113
AFTR	LC	**0.749**	**0.732**	0.277	0.239	0.332	0.206	0.114

The best result for each column is in bold.

**Table 2:** Comparison of AFTR with various SOTA methods on nuScenes validation set. Abbreviations: C is cameras, L is LiDAR, and LC is LiDAR and cameras. “FUTR3D-C” denotes a model trained and tested using only camera data, and so on.

Methods	Modality	NDS	mAP
UVTR-L [39]	L	0.676	0.608
TransFusion-L [37]	L	0.702	0.655
FUTR3D-L [15]	L	0.655	0.593
CMT-L [13]	L	0.686	0.624
UVTR [39]	LC	0.702	0.654
TransFusion [37]	LC	0.713	0.675
FUTR3D [15]	LC	0.683	0.645
CMT [13]	LC	0.729	0.703
BEVFusion [5]	LC	0.714	0.685
DeepInteraction [38]	LC	0.726	0.703
AFTR-L	L	0.699	0.636
AFTR	LC	**0.735**	**0.704**

The best result for each column is in bold.

**Table 3:** Ablation results of AFTR with different image sizes as input data on nuScenes validation set.

Image Size	NDS	mAP	mATE	mASE	mAOE	mAVE	mAAE
800 × 320	0.708	0.658	**0.280**	0.252	0.331	0.230	0.121
1600 × 640	**0.735**	**0.704**	0.283	**0.247**	**0.313**	**0.212**	**0.115**

The best result for each column is in bold.

**Table 4:** Ablation results of AFTR with different backbones as image feature extractors on nuScenes validation set.

Backbone	NDS	mAP	mATE	mASE	mAOE	mAVE	mAAE
ResNet-50	0.704	0.676	**0.282**	**0.241**	0.387	0.301	0.128
ResNet-101	0.725	0.695	0.301	0.250	0.334	0.222	0.117
VoVNet-99	**0.735**	**0.704**	0.283	0.247	**0.313**	**0.212**	**0.115**

The best result for each column is in bold.

**Table 5:** Ablation results of AFTR with different voxel sizes on nuScenes validation set.

Voxel Size	NDS	mAP	mATE	mASE	mAOE	mAVE	mAAE
0.075 m	**0.735**	**0.704**	**0.283**	**0.247**	0.313	**0.212**	**0.115**
0.100 m	0.727	0.689	0.285	0.249	**0.311**	0.214	0.117
0.125 m	0.710	0.661	0.294	0.249	0.323	0.218	0.120

The best result for each column is in bold.

**Table 6:** Ablation results of different point cloud data representations on the nuScenes validation set. The BEV representation is obtained by compressing the 3D voxel features along the z axis, and then ASCA interacts with LiDAR features in the same way as with image features.

Representation	NDS	mAP	mATE	mASE	mAOE	mAVE	mAAE
BEV	0.727	0.695	0.288	0.251	0.315	0.213	0.124
3D	**0.735**	**0.704**	**0.283**	**0.247**	**0.313**	**0.212**	**0.115**

The best result for each column is in bold.

**Table 7:** Ablation results of nuScenes validation set with or without AFTR using temporal data. AFTR-s indicates that STSA is not used to interact with the history query, and vanilla self-attention [9] is used to interact with the input query itself.

Temporal	NDS	mAP	mATE	mASE	mAOE	mAVE	mAAE
AFTR-s	0.691	0.663	0.286	0.250	**0.312**	0.437	0.118
AFTR	**0.735**	**0.704**	**0.283**	**0.247**	0.313	**0.212**	**0.115**

The best result for each column is in bold.

**Table 8:** Ablation results of the number of offsets in AFTR. The ASCA and STSA modules are implemented by deformable attention [26] and sample and interact with features based on the offset positions of the projection points, with the number of offsets $N_{offset}$. $N_{offset}=0$ is where the query interacts only with the feature at the projection position, and global is where it interacts with all the features on the feature map using vanilla attention [9].

$N_{offset}$	NDS	mAP	mATE	mASE	mAOE	mAVE	mAAE
0	0.721	0.688	0.314	0.251	0.317	0.225	0.120
4	**0.735**	0.704	0.283	**0.247**	**0.313**	**0.212**	**0.115**
8	0.735	**0.706**	**0.282**	0.249	0.315	0.220	0.117
Global	0.698	0.657	0.342	0.266	0.324	0.231	0.138

The best result for each column is in bold.

**Table 9:** The robustness studies of AFTR under misalignment on the nuScenes validation set. *n* denotes noise level, the method tail “-a” denotes a model retrained using noisy data, “-g” denotes a model using vanilla attention [9] instead of deformable attention, and “-s “ denotes models that do not use temporal data as mentioned in Section 5.2.4. For models that are not trained with noisy data, we generated results by only using the validation set that is disturbed by noise.

Methods	*n* = 0		*n* = 1		*n* = 2		*n* = 3		*n* = 4		Delta
	NDS	mAP	NDS	mAP	NDS	mAP	NDS	mAP	NDS	mAP	
FUTR3D [15]	0.683	0.645	0.652	0.613	0.616	0.581	0.596	0.525	0.554	0.515	0.189
FUTR3D-a	0.676	0.633	0.644	0.609	0.607	0.562	0.592	0.541	0.551	0.508	0.185
BEVFormer [9]	0.517	0.416	0.512	0.414	0.495	0.392	0.473	0.375	0.447	0.352	0.135
BEVFormer-a	0.506	0.407	0.503	0.405	0.492	0.396	0.476	0.385	0.457	0.366	0.097
AFTR-sg	0.654	0.611	0.651	0.608	0.648	0.599	0.641	0.592	0.633	0.582	0.032
AFTR-s	0.691	0.663	0.687	0.656	0.664	0.639	0.632	0.583	0.597	0.562	0.136
AFTR-sa	0.697	0.665	0.696	0.660	0.682	0.644	0.664	0.623	0.634	0.599	0.091
AFTR	**0.735**	**0.704**	**0.733**	**0.699**	**0.711**	**0.676**	0.676	0.644	0.642	0.607	0.127
AFTR-a	0.721	0.688	0.720	0.688	0.709	0.673	**0.688**	**0.653**	**0.659**	**0.611**	**0.086**

The best result for each column is in bold.

## Data Availability

The dataset generated and analyzed during the current study is available in the nuScenes repository (https://www.nuscenes.org/nuscenes, accessed on 29 August 2023).
